# A new Burgess Shale-type deposit from the Ediacaran of western Mongolia

**DOI:** 10.1038/srep23438

**Published:** 2016-03-18

**Authors:** Stephen Q. Dornbos, Tatsuo Oji, Akihiro Kanayama, Sersmaa Gonchigdorj

**Affiliations:** 1 Department of Geosciences, University of Wisconsin-Milwaukee, Milwaukee, WI 53211, USA; 2 Nagoya University Museum, Nagoya University, Nagoya 464-8601, Japan; 3 Mongolian University of Science and Technology, Ulaanbaatar 46/520, Mongolia

## Abstract

Preservation of soft-bodied organisms is exceedingly rare in the fossil record. One way that such fossils are preserved is as carbonaceous compressions in fined-grained marine sedimentary rocks. These deposits of exceptional preservation are known as Burgess Shale-type (BST) deposits. During the Cambrian Period, BST deposits are more common and provide a crucial view of early animal evolution. The earliest definitive fossil evidence for macroscopic animal-grade organisms is found in the preceding Ediacaran Period. BST deposits from the Ediacaran are rarer and lack conclusive evidence for animals. Here we report the discovery of a new Ediacaran BST deposit with exceptional preservation of non-mineralizing macro-organisms in thinly bedded black shale from Zavkhan Province, western Mongolia. This fossil assemblage, here named the Zuun-Arts biota, currently consists of two new species of probable macroscopic multicellular benthic algae. One species, *Chinggiskhaania bifurcata* n. gen., n. sp., dominates the biota. The other species, *Zuunartsphyton delicatum* n. gen., n. sp., is known from three specimens. SEM-EDS analysis shows that the fossils are composed of aluminosilicate clay minerals and some carbon, a composition comparable to fossils from the Cambrian Burgess Shale biota. This discovery opens a new window through which to view late Precambrian life.

Ediacaran BST deposits are known from several localities around the globe, including Siberia^1^, India^2^, Paraguay^3^, the western United States^4^, the Yangtze Platform of South China^5^,^6^, and various upper Ediacaran deposits containing the multicellular benthic alga *Vendotaenia*^7^,^8^. Most of these assemblages are low-diversity and preserve multicellular benthic algae and/or enigmatic fossil taxa. None of them contain unambiguous animal fossils.

The most diverse of these assemblages are the two from the Yangtze Platform of South China, the Lantian and Miaohe biotas^5^,^6^. They both contain mostly benthic multicellular eukaryotic algae fossils preserved as carbonaceous compressions in fine-grained marine shales^5^,^6^,^9^,^10^. They share several algal taxa^10^. The Lantian biota is preserved in the Ediacaran Lantian Formation in Anhui Province, China, and contains 15 taxa, some of which have been interpreted as cnidarian, or even potentially bilaterian-grade, organisms^9^. The Miaohe biota contains about 20 taxa and is preserved in the upper part of the Ediacaran Doushantuo Formation in Hubei Province, China^6^. It contains one possible vermiform animal taxon^6^. The age of the Miaohe biota is more constrained, ranging from 591−551 Ma^11^. The older of these radiometric dates remains controversial, however^12^.

Abundant fragmentary specimens of a new species of probable benthic multicellular algae, *Chinggiskhaania bifurcata* n. gen., n. sp., dominates the Zuun-Arts biota. The biota is preserved in black shales near the base of the upper Ediacaran to Cambrian Zuun-Arts Formation^13^ in the Zavkhan Basin of Zavkhan Province of western Mongolia (Fig. 1). Carbonates dominate the formation in its Ediacaran portion and it underlies the mixed carbonate–siliciclastic sequences of the Cambrian Bayan Gol Formation. The regional Ediacaran–Cambrian boundary is placed near the top of the Zuun-Arts Formation based on a large negative carbon isotope excursion^14^ and the first appearance of the penetrative trace fossil *Treptichnus pedum* in the overlying Bayan Gol Formation^14^,^15^.

## Results

**Figure 1:**
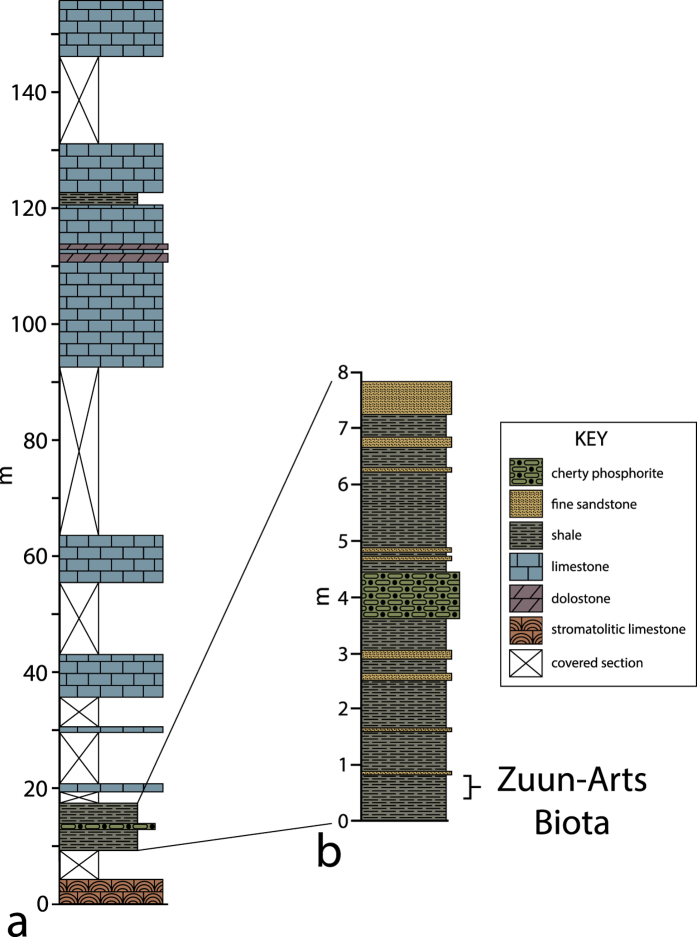
Stratigraphic context of Zuun-Arts biota. (**a**) Stratigraphic column of the Zuun-Arts Formation at the fossil locality. (**b**) Detailed stratigraphic column of the rock units containing the Zuun-Arts biota at the base of the Zuun-Arts Formation. Initial excavation of the Zuun-Arts biota has focused on in an interval from 40 to 80 cm within the basal black shale. Exceptionally preserved algal fossils have also been recovered from talus higher in the shale. These strata will be excavated in the future. Figure was drafted using Adobe Illustrator CC (www.adobe.com/illustrator).

The Zuun-Arts biota is preserved in the Zuun-Arts region of Zavkhan Province, between the cities of Uliastai to the north and Altai to the south. The fossils are preserved in a thinly bedded black shale facies that is slightly less than 7 m thick (Fig. 1). The fossiliferous zone is in strata from 40 cm to 80 cm within the black shale (Fig. 1). Petrographic thin section analysis shows the dominant microfacies to be an unmetamorphosed thinly laminated quartzose siltstone (Supplementary Fig. 1). The fossils are found between two distinctive facies: a lower carbonate containing conspicuous stromatolites and an upper thin bed of cherty phosphorite (Fig. 1). These bounding facies are correlative to stratigraphic units 9 and 10 of Khomentovsky and Gibsher^16^ at the Ediacaran–Cambrian boundary section at Bayan Gol in adjacent Gobi-Altai Province. The biota is therefore at the base of the Zuun-Arts Formation^13^,^14^,^17^ in what appears to be a transgressive lag deposit. Recent sequence stratigraphic, chemostratigraphic, and biostratigraphic work confirms an upper Ediacaran age for these strata^13^,^14^,^17^.

This biota’s age relationship to the diverse Lantian and Miaohe biotas remains unclear and it is not currently possible to determine its absolute age. The oldest convincing Ediacaran trace fossils date to about 555 Ma^18^, and this biota occurs below the earliest trace fossils in this region^13^. This may mean that it is slightly older than 555 Ma, which would put it within the younger age estimates for the Miaohe biota^11^.

**Figure 2:**
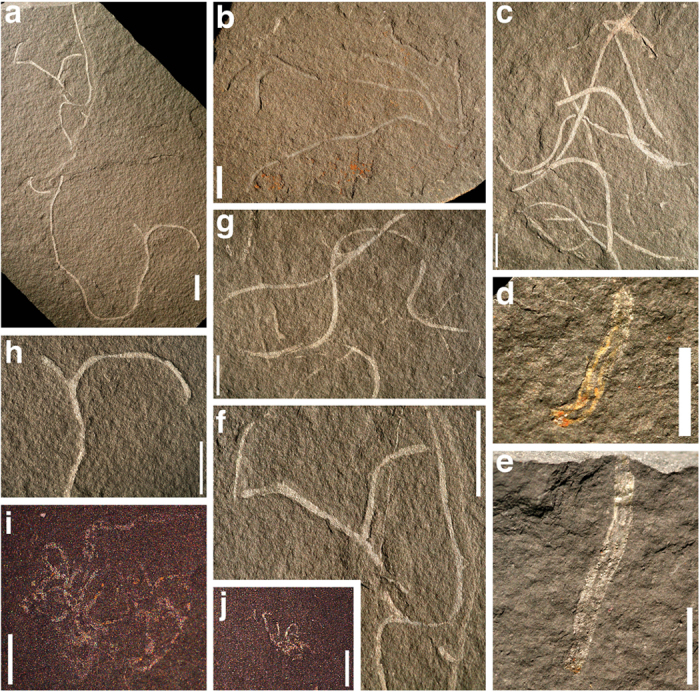
Photographs and photomicrographs of exceptionally preserved multicellular algae fossils from Zuun-Arts biota. (**a–h**) *Chinggiskhaania bifurcata*. Photographs taken under cross-polarized light. Scale bars = 5 mm. (**a**) Specimen showing characteristic thin, rarely branching filaments (IEZAB0002). (**b**) Specimen of a thallus containing four filaments, one branching, that converge toward the lower right of the photograph (holotype; IEZAB0001). Base of specimen is poorly preserved. (**c**) Fragmentary filaments preserved in a bundle (IEZAB0003). (**d**) Basal portion of a thallus showing stipe comprised of closely grouped filaments above a narrow holdfast (IEZAB0004). (**e**) Another specimen of basal portion of a thallus from the same slab as 2d showing stipe and holdfast (IEZAB0004). (**f–h**) Typical branching of filaments (IEZAB0002, IEZAB0003, and IEZAB0002, respectively). (**i,j**) *Zuunartsphyton delicatum*. Photomicrographs taken under normal light. Scale bars = 1 mm. (**i**) Larger specimen showing shrub-like thallus with curly filaments (holotype; IEZAB0007). (**j**) Smaller specimen also showing shrub-like thallus with curly filaments (IEZAB0008).

*Chinggiskhaania bifurcata* has thin filaments lacking transverse longitudinal divisions or ornamentation that gently curl and rarely branch (Fig. 2a). The filaments have fine lengthwise lineations that are not always well preserved. Mean filament width is 0.47 mm (N = 100) and ranges from 0.23 mm to 0.76 mm (STDEV = 0.12 mm). There does not appear to be any consistent distal tapering in filament width. Some filaments have fluctuating widths along their length, suggestive of twisting deformation. The filament branching angle ranges from 43° to 85° with a mean of 63° and standard deviation of 15° (N = 7). Filament fragments are the most common mode of preservation and they are innumerable in the fossiliferous zone of the strata. One well-preserved specimen contains just four filaments, showing that the filaments are not densely grouped (Fig. 2b). Two specimens preserve details of the basal region of the organism, showing a narrow attachment area below a stem of tightly gathered filaments (Fig. 2d,e). Based on the branching filaments, thallus-like morphology, and basal attachment structures that resemble a stipe and holdfast, *C. bifurcata* is interpreted as a multicellular benthic alga. *C. bifurcata* is most comparable to *Doushantuophyton* from the Miaohe biota and *Huangshanophyton* from the Lantian biota^6^,^9^. It differs significantly from these genera in that its filaments are not as densely assembled in the thallus as either genus, they do not branch as commonly as in *Doushantuophyton*, and they lack the septation of the filaments of *Huangshanophyton*^6^,^9^.

The other new species of probable multicellular benthic algae in this biota, *Zuunartsphyton delicatum* n. gen., n. sp., is known from three individual specimens. It has a small shrub-like morphology, less than 3 mm in diameter, composed of thin tightly curling filaments (< 0.1 mm wide) that do not branch and lack transverse longitudinal divisions or ornamentation (Fig. 2i,j). Its attachment structures are unknown. *Z. delicatum* is interpreted as a multicellular benthic algae species because of its thallus-like morphology composed of thin filaments. This species is not closely comparable to any algal taxa from the Miaohe or Lantian biotas.

**Figure 3:**
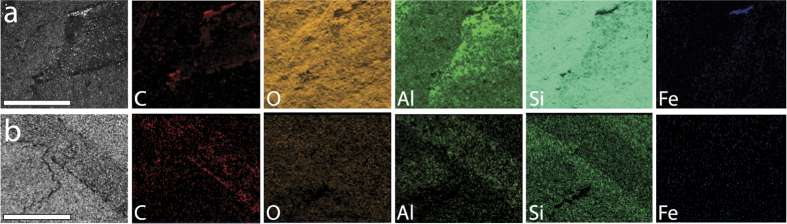
SEM-EDS images of *Chinggiskhaania bifurcata* filaments. (**a**) One filament and its EDS images. Original backscatter image on left. Scale bar is 600 μm. Note high concentrations of Al, Si, and some C in filament. There is also one small zone of high Fe concentration shown in blue. (**b**) One filament and its EDS images. Original backscatter image on left. Scale bar is 600 μm. Note high concentrations of Al, Si, and some C in filament.

SEM-EDS analysis of two *C. bifurcata* filaments shows consistent high concentrations of Al and Si relative to other elements, although Si is not enriched relative to the matrix (Fig. 3). C is also locally concentrated in portions of the specimens, but is not preferentially associated with any morphological feature of the fossils (Fig. 3). These results are consistent with preservation as aluminosilicate clay mineral films with some carbon present. The fossils were likely originally preserved as carbon films and were diagenetically altered to aluminosilicate minerals. Small areas of high Fe concentration also occur in one specimen in the same zones as high C concentration (Fig. 3a). SEM examination of these zones reveals framboidal minerals consistent with pyrite (Supplementary Fig. 2). This pyrite likely precipitated as a result of the sulfate reduction during the decay of the filaments. Similar pyrite framboid concentrations are known from Lantian and Miaohe biota fossils^10^. These SEM-EDS results are comparable to the fossil assemblages of the middle Cambrian Burgess Shale biota, which are also preserved as aluminosilicate clay mineral films^19^. This preservational style contrasts with the Lantian and Miaohe biotas, and most Cambrian Burgess Shale-type deposits, which primarily preserve fossils as carbon films^10^,^20^.

## Discussion

Discovery of the exceptionally preserved fossils of the Zuun-Arts biota opens a new preservational window through which to view Ediacaran multicellular life. It is currently a low-diversity assemblage consisting of two probable multicellular benthic algae species. In this way, the Zuun-Arts biota is comparable to most other Ediacaran BST biotas, which are either monospecific or low-diversity assemblages of probable multicellular benthic algae or enigmatic taxa. Future excavation of this biota will determine if this remains the case.

At this point, the Zuun-Arts biota is also similar to all other Ediacaran BST deposits in that it contains no unambiguous evidence for animals. Macroscopic animal-grade organisms are well known from other Ediacaran taphonomic windows, most notably in classical Ediacaran biota-style sandstone mold and cast preservational settings. With their sub-millimeter preservational capabilities, Ediacaran BST deposits should theoretically preserve animals relatively easily. This is certainly true of Cambrian BST deposits, which preserve a range of soft-bodied animal phyla in exquisite detail^21^. Although putative animal fossils have been described from Ediacaran BST deposits^6^, it remains unclear why they do not contain clear animal fossils. One possible explanation may be that the kind of animals that this preservational mode favors, such as ecdysozoans with their more preservable recalcitrant cuticle tissues^22^, simply did not exist yet.

## Methods

The Zuun-Arts biota was discovered in the summer of 2014 from loose fossils in float. Initial excavation began in the summer of 2015 and a total of 77 *in situ* specimens have been collected from strata between 40 cm and 80 cm in the section (Fig. 1). Numerous poorly preserved fragmentary specimens were left behind. Morphological data was collected in the laboratory, consisting of 100 filament width measurements and 7 branching angle measurements. All fossil specimens will be permanently reposited at the Museum of Geology and Mineral Resources of the Mongolian University of Science and Technology (MUST). They have been assigned permanent International Geo Sample Numbers (IGSN) with the prefix “IEZAB-”. The System for Earth Sample Registration (http://www.geosamples.org) administers IGSNs and they are searchable online. *Chinggiskhaania bifurcata* fossils were photographed with a Canon EOS Digital Rebel XT on a camera stand under cross-polarized light. *Zuunartsphyton delicatum* fossils were photographed under normal light with a Zeiss AxioCam MRc5 digital imager attached to a Zeiss Stemi 2000-C binocular microscope. Zeiss Axiovision digital imaging software was used to process these images. SEM-EDS analysis was performed with a Hitachi S-3400N scanning electron microscope.

## Additional Information

**How to cite this article**: Dornbos, S. Q. *et al.* A new Burgess Shale-type deposit from the Ediacaran of western Mongolia. *Sci. Rep.* **6**, 23438; doi: 10.1038/srep23438 (2016).

## Supplementary Material

Supplementary Information
