# Supplementary material for: A new Burgess Shale-type deposit from the Ediacaran of western Mongolia
Source: Sci Rep. 2016 Mar 18;6:23438. doi: 10.1038/srep23438 (PMC4796905; doi:10.1038/srep23438)
Supplement: Supplementary Information [file srep23438-s1.pdf]

Title: A new Burgess Shale-type deposit from the Ediacaran of western Mongolia

Authors: Stephen Q. Dornbos[1*], Tatsuo Oji[2], Akihiro Kanayama[2], Sersmaa Gonchigdorj[3]

Affiliations:

[1]Department of Geosciences, University of Wisconsin-Milwaukee, Milwaukee, WI 53211, USA

[2]Nagoya University Museum, Nagoya University, Nagoya 464-8601, Japan

[3]Mongolian University of Science and Technology, Ulaanbaatar 46/520, Mongolia

[*]Correspondence to: sdornbos@uwm.edu

Supplementary Information:

Figure 1

Figure 2

Systematic Palaeontology

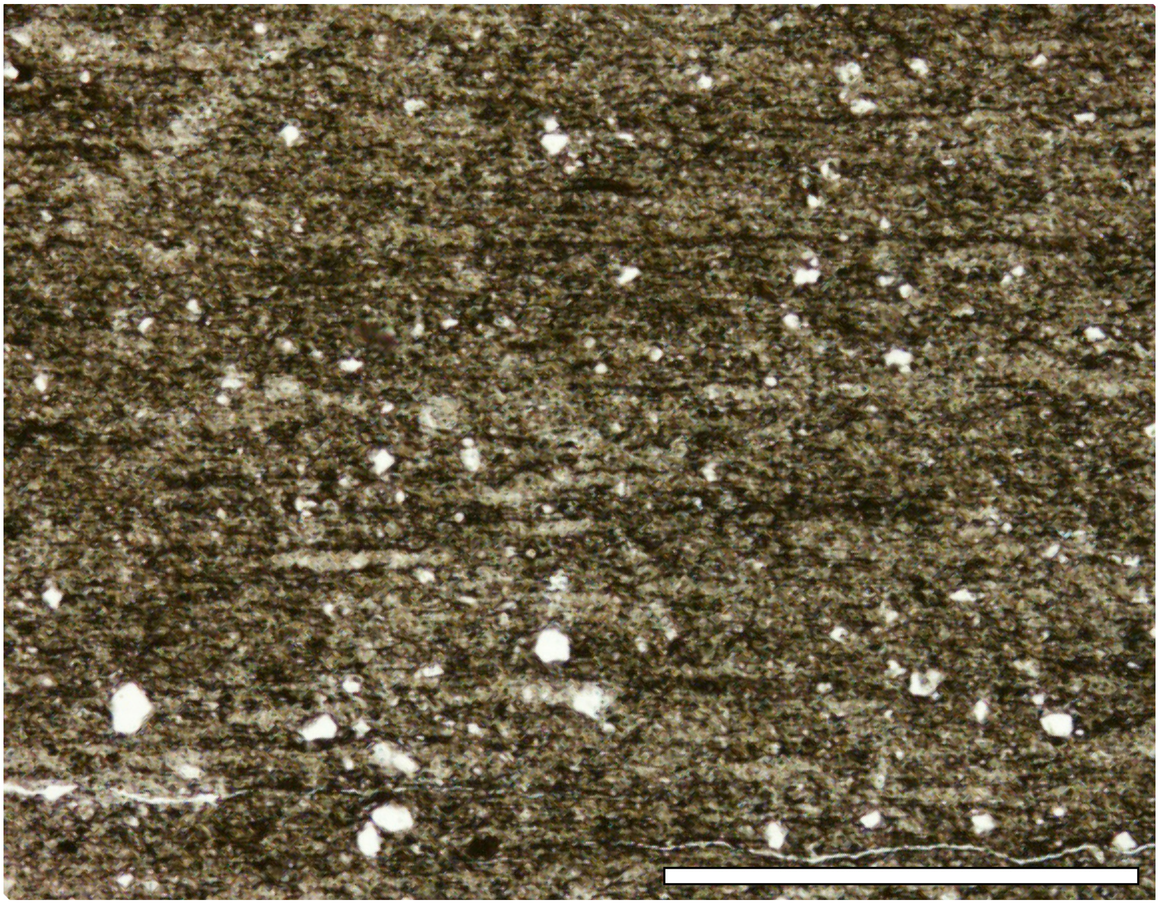

Figure 1 | Photomicrograph of dominant microfacies in which Zuun-Arts biota is preserved. An unmetamorphosed thinly laminated quartzose siltstone. Large grains are quartz. Scale bar = 0.5 mm.

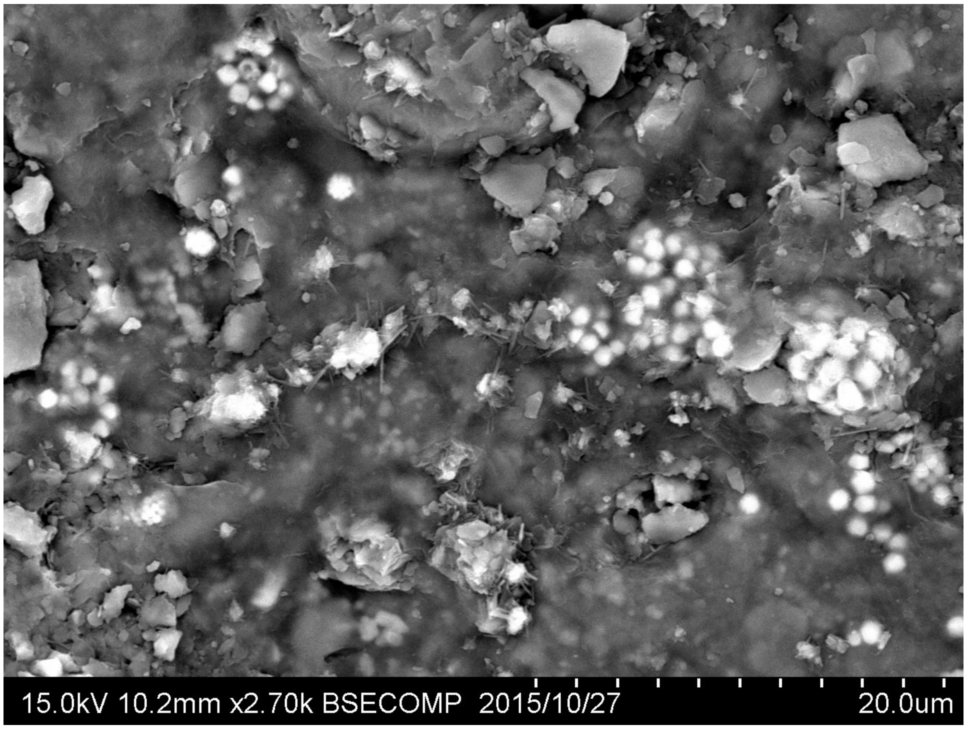

Figure 2 | SEM photomicrograph of framboidal pyrite from high Fe concentration zone of *Chinggiskhaania bifurcata* filament.

Systematic Palaeontology

The higher taxonomy of these genera within the macrophytes is unclear because they are only known from fossil remains. Specimens will be permanently reposited at the Museum of Geology and Mineral Resources of the Mongolian University of Science and Technology (MUST). They have been assigned permanent International Geo Sample Numbers (IGSN) with the prefix “IEZAB-“. The System for Earth Sample Registration (http://www.geosamples.org) administers IGSNs and they are searchable online.

Genus CHINGGISKHAANIA new genus

Type species.- *Chinggiskhaania bifurcata* new species.

Diagnosis.- Has thin filaments lacking transverse longitudinal divisions or ornamentation that gently curl and rarely branch. The filaments have fine lengthwise lineations that are not always well preserved. Mean filament width is 0.47 mm (N = 100) and ranges from 0.23 mm to 0.76 mm (STDEV = 0.12 mm). There does not appear to be any consistent distal tapering in filament width. Some filaments have fluctuating widths along their length, suggestive of twisting deformation. The filament branching angle ranges from 43° to 85° with a mean of 63° and standard deviation of 15° (N = 7). One well-preserved specimen contains just four filaments, showing that the filaments are not densely grouped. Two specimens preserve details of the basal region of the organism, showing a narrow attachment area below a stem of tightly gathered filaments.

Etymology.- After Chinggis Khaan, the founder of the Mongol Empire and Great Khaan from 1206–1227. References the preservation of this genus in Mongolia.

Discussion.- Based on the branching filaments, thallus-like morphology, and basal attachment structures that resemble a stipe and holdfast, *Chinggiskhaania* is interpreted as a multicellular benthic alga. Its morphological characters differentiate it from other published Ediacaran algae genera. See below for further discussion.

CHINGGISKHAANIA BIFURCATA new species

Figure 2a – 2h

Diagnosis.- As per genus.

Description.- Preserved as two-dimensional aluminosilicate clay mineral films with some carbon in a laminated black shale. Thallus comprised of gently curling, thin filaments (mean width = 0.47 mm) that rarely branch dichotomously (mean branching angle = 63°). Filaments lack septation and converge to form a stipe that descends into a narrow attachment holdfast. Holotype has a thallus with four filaments and an overall thallus length of less than 5 cm.

Etymology.- From the Latin *bifurcata*, meaning “forked in two”. References the branching nature of its filaments.

Type.- The specimen illustrated in Figure 2b is designated the holotype, as it is the most well preserved thallus (IEZAB0001).

Material.- Six specimens (IEZAB0001, IEZAB0002, IEZAB0003, IEZAB0004, IEZAB0005, IEZAB0006). Four of these specimens are illustrated. Fragmentary specimens are too numerous to count or collect at the fossil locality.

Occurrence.- Ediacaran black shales in the lower Zuun-Arts Formation, southern Zavkhan Province, Mongolia.

Discussion.- Two Ediacaran algae taxa are generally comparable to this new species: *Doushantuophyton* from the Miaohe biota and *Haungshanophyton* from the Lantian biota[1,2]. This new species differs significantly from these genera in that its filaments are not as densely grouped in the thallus as either genus, they do not branch as commonly as in *Doushantuophyton*, and they lack the septation of the filaments of *Huangshanophyton*[1,2].

Genus ZUUNARTSPHYTON new genus

Type species.- *Zuunartsphyton delicatum* new species

Diagnosis.- Has a small shrub-like morphology, less than 3 mm in diameter, composed of thin tightly curling filaments (<0.1 mm wide) that do not branch and lack transverse longitudinal divisions or ornamentation. Its attachment structures are unknown.

Etymology.- After the Zuun-Arts Formation, in which it is preserved. From the Latin *phyton*, meaning "plant."

Discussion.- Interpreted as a multicellular benthic algae species because of its thallus-like morphology composed of thin filaments. This genus does not closely resemble any other published Ediacaran algae genera. See below for further discussion.

ZUUNARTSPHYTON DELICATUM new species

Figure 2i – 2j

Diagnosis.- As per genus.

Description.- Preserved as two-dimensional aluminosilicate clay mineral films with some carbon in a laminated black shale. Tiny shrub-like thallus (< 3 mm in diameter) with numerous

thin curly filaments (< 0.1 mm wide). Filaments do not branch and are unseptated. Stipe and holdfast structures, if present, are currently unknown.

Etymology.- From the Latin *delicatum*, meaning “delicate”. References the small size of the thallus and its thin filaments.

Type.- The specimen illustrated in Figure 2i is designated the holotype (IEZAB0007).

Material.- Two specimens (IEZAB0007and IEZAB0008). Both of these specimens are illustrated, but one of them (IEZAB0007) contains two thalli, one of which is not illustrated.

Occurrence.- Ediacaran black shales in the lower Zuun-Arts Formation, southern Zavkhan Province, Mongolia.

Discussion.- This new species is not closely comparable to other published Ediacaran algae species and the filaments lack any structures consistent with the cells of cyanobacteria. This species is so small that in a modern setting it might be considered part of the periphyton, a biofilm community of eukaryotic algae, cyanobacteria, other microorganisms, and microinvertebrates that is ubiquitous on aquatic hard substrates[3]. It is unknown if such complex associations existed in the Ediacaran.

References

1. Xiao, S., Yuan, X. L., Steiner, M. & Knoll, A. H. Macroscopic carbonaceous compressions in a terminal Proterozoic shale: A systematic reassessment of the Miaohe biota, South China. *J. Paleontology* **76**: 347–376 (2002).

2. Yuan, X. L., Chen, Z., Xiao, S., Zhou, C. & Hua, H. An early Ediacaran assemblage of macroscopic and morphologically differentiated eukaryotes. *Nature* **470**: 390–393 (2011).

3. Sanli, K., Bengtsson-Palme, J., Nilsson, R. H., Kristiansson, E., Rosenblad, M. A., Blanck, H. & Eriksson, K. M. Metagenomic sequencing of marine periphyton: Taxonomic and functional insights into biofilm communities. *Front. Microbial.* **6**: 1192.
